# Side effects of chronic systemic glucocorticoid therapy: what dermatologists should know^☆^

**DOI:** 10.1016/j.abd.2023.05.005

**Published:** 2023-11-24

**Authors:** Lucas Campos Prudente Tavares, Lívia de Vasconcelos Nasser Caetano, Mayra Ianhez

**Affiliations:** Department of Tropical Medicine and Dermatology, Hospital das Clínicas da Universidade Federal de Goiás, Goiânia, Goiás, Brazil

**Keywords:** Glucocorticoids, Drug-related side effects and adverse reactions, Drug monitoring, Risk factors

## Abstract

In dermatologists' clinical practice, the use of systemic glucocorticoids is recurrent for the management of different comorbidities that require chronic immunosuppression. The prescription of this medication requires caution and basic clinical knowledge due to the several adverse effects inherent to the treatment. However, different doubts may arise or inappropriate conduct may be adopted due to the lack of objective and specific guidelines for the screening, prophylaxis and management of complications from chronic corticosteroid therapy. Considering this problem, the authors carried out a narrative review of the literature to gather up-to-date data on adverse effects secondary to the chronic use of systemic glucocorticoids. The broad approach to this topic made it possible to review the pathophysiology and risk factors for these complications, as well as to develop updated orientation that can be used as a learning tool and quick reference for dermatologists during their clinical practice with glucocorticoids.

## Introduction

Glucocorticoids (GCs) are medications indicated for the treatment of numerous diseases due to their anti-inflammatory and immunosuppressive properties. They can be prescribed by general physicians in primary care centers for the treatment of common diseases, and by specialists for the outpatient management of more complex diseases, for hospitalized patients or the critically ill ones in intensive care units. GCs have been frequently and widely used in medicine since the 1950s, after the first reports demonstrating their effectiveness in the treatment of rheumatoid arthritis. The discovery of this class of medication was so revolutionary for science that Edward Kendall, Tadeusz Reichstein and Philip Hench received the Nobel Prize in Medicine and Physiology after they isolated cortisone, which allowed its medical use.^1^, ^2^

Despite their recognized benefits, their use can lead to serious adverse effects in different organs, and this risk can reach 90% in patients who use it for more than 60 days. Adverse effects are quite variable and depend on treatment duration, route of administration, and can occur even with low doses of GCs.^1^ Weight gain, hyperglycemia, diabetes mellitus (DM), adrenal suppression, osteoporosis, dermatological changes, cardiovascular complications, cataracts, glaucoma, peptic ulcer, myopathy, increased propensity to infections and neuropsychiatric disorders are the main possible adverse effects reported in the literature for patients undergoing chronic systemic corticosteroid therapy. The physician should be aware of these adverse effects from the start of treatment in order to minimize the impact of these medications on the patient's health.^1^, ^3^, ^4^

## Objective

In the daily practice of dermatology, many clinical conditions demand the chronic use of GCs, especially bullous dermatoses and autoimmune diseases. When the chronic use of this medication is chosen, it is imperative that the prescriber be aware of the different adverse effects, their risk factors, as well as the prophylactic measures, treatment, and the need for monitoring together with other specialists. The objective of this study is to present a narrative review of the literature that depicts the main adverse effects of these drugs and helps answer three main questions that a dermatologist may have when managing a patient on chronic systemic corticosteroid therapy: what are the main risk factors for a given complication? Are there prophylactic measures? How often should one screen for such complications?

## Method

For this narrative literature review, the MEDLINE scientific database was used through the PubMed search tool. Articles published between 2015 and 2021 and indexed with the MeSH terms “Glucocorticoids [MeSH] AND Glucocorticoids/adverse effects [Majr]” were searched. This resulted in a total of 1,244 articles. Then, the authors read the abstracts, one by one, and 89 articles were pre-selected for full reading. Articles that addressed the use of glucocorticoids by routes other than the systemic one, articles focused on specific diseases or articles that were little related to the use of GCs were excluded. Then, of the 89 pre-selected articles, those written in languages other than English or Portuguese, articles unavailable for full reading, and essays and reviews focused on very specific treatments were excluded. The selection process was carried out jointly by the authors without any disagreement. Finally, 38 articles were included in this review.

## Definitions

As for treatment duration, treatment can be considered short if it lasts less than three months, long or chronic when it lasts more than six months, and of intermediate duration if it lasts between three and six months. The potency of a systemic GC can be defined according to its affinity for its intracellular receptor and the time of action. Among the main drugs used in clinical practice, hydrocortisone is short-acting (8‒12 hours), prednisone, prednisolone, methylprednisolone and triamcinolone are intermediate-acting (12‒36 hours), whereas betamethasone and dexamethasone are long-acting (36‒72 hours).^3^ The group of experts from the European League Against Rheumatism (EULAR) reinforces that the level of harm caused by the chronic use of GCs is directly associated with the dose and each patient individual characteristics. Patients who make chronic use of GCs at a daily dose equivalent to ≤5 mg of prednisone were established as having a low risk for adverse effects, and at high risk if the daily dose is ≥10 mg. At intermediate daily doses, between 5 and 10 mg, the risk depends on specific patient characteristics.^4^

## Hyperglycemia and DM

GCs exert an important effect on carbohydrate metabolism. They act directly on pancreatic β-cells, reducing their viability and inhibiting insulin secretion. In peripheral tissues, GCs increase insulin resistance and, in the liver, they stimulate gluconeogenesis. Thus, they favor weight gain, cause an important hyperglycemic state, and induce or worsen DM.^1^, ^5^, ^6^

GC-induced hyperglycemia may appear as early as the second week.^6^ It is marked by increased serum glucose levels, especially postprandial blood glucose, which is one of the main indicators of GC-induced DM.^7^ The main risk factors associated with GC-induced hyperglycemia/DM are: obesity, advanced age, genetic predisposition, and chronic inflammation.^2^, ^4^, ^7^ Smoking, high blood pressure, and high levels of glycated hemoglobin have also been cited as risk factors.^8^

It is crucial that patients using GCs be advised by their physicians about the risk of hyperglycemia and the signs/symptoms of suspected DM (polyuria, polydipsia, polyphagia). Emphasis should be placed on changing lifestyle habits, which include healthy diets, weight loss, and physical activity.^4^, ^7^ Monitoring the patient's blood glucose is mandatory and should be done alongside the family doctor or endocrinologist. Although there are no specific recommendation in the literature regarding the frequency of such monitoring, checking glycated hemoglobin before treatment should be considered and, if altered, further investigation of DM must be carried out, thus reinforcing the need for monitoring throughout treatment.^7^ It is also recommended that the patient routinely test for capillary blood glucose, especially in the postprandial period.^6^ Caplan et al. recommend that patients check capillary blood glucose in the afternoon, two to three times a week. Levels >200 mg/dL are a warning for new therapeutic measures.^7^ In this review, no specific data or guidelines were found to determine how often laboratory screening for DM should be performed during treatment with GCs. In the authors' clinical practice, screening is performed in the first month and then at least every 6 months, due to the insidious nature of insulin resistance development. Table 1 summarizes the main considerations for GC-induced DM.

**Table 1:** Main risk factors, prophylaxis and monitoring for glucocorticoid-induced diabetes mellitus.

Main risk factors	Obesity, advanced age, genetic predisposition, chronic inflammation, smoking, high blood pressure, high levels of glycated hemoglobin.
Prophylactic measures:	• Healthy diet and physical activity.
	• Metformin can be helpful.
Monitoring:	• Glycated hemoglobin at the start of treatment.
	• Laboratory screening at least every 6 months.
	• Assessment of signs and symptoms of DM.
	• Capillary blood glucose in the afternoon at least 2 to 3 times a week.
	• Joint follow-up with an endocrinologist if necessary.

DM: diabetes mellitus.

Different medications are available for the treatment of GC-induced hyperglycemia, but there are few recommendations to determine the best choice, and therefore the patient should be treated together with the general physician or endocrinologist.^7^ Many authors consider insulin as the treatment of choice.^6^, ^8^ Other antidiabetic drugs, such as metformin, sulfonylureas, thiazolidinediones, DPP-4 inhibitors, and GLP-1 agonists, have shown some efficacy in some studies, although for some authors these drugs would not be as effective due to the significant insulin resistance imposed by the GCs.^6^, ^9^ In a randomized clinical trial, the authors demonstrated that the use of metformin can optimize glycemic control during GC treatment. Non-diabetic patients who would undergo the use of GCs at doses >7.5 mg/day for at least four weeks were evaluated. The use of metformin at a dose of 850 mg/day for one week, followed by 850 mg twice a day for three weeks, showed superior glycemic control of these patients when compared to the placebo group, confirmed by laboratory tests after four weeks of treatment (fasting blood glucose, oral glucose tolerance test, HOMA index, and fasting insulin). Despite the small number of patients studied, these results represent important data for future measures regarding the prevention of DM during corticosteroid therapy.^10^

## Adipose tissue changes, weight gain and Cushing syndrome

In adipose tissue, GCs exert both a lipolytic and lipogenic effect, being responsible for increasing free fatty acids, reducing subcutaneous fat, and stimulating visceral fat. These changes lead to body fat redistribution, induce dyslipidemia, and increase cardiovascular risk.^1^, ^5^, ^6^

There are few data regarding GC-induced dyslipidemia and specific guidelines regarding its treatment are lacking. Therefore, preventive measures and treatment are the same as in general clinical practice.^6^

Weight gain in chronic use of GCs, although little studied, seems to be mainly associated with dose and treatment duration. Other risk factors are female gender, young age, and smoking.^11^ Despite being underestimated by physicians, weight gain was considered one of the most relevant adverse effects by patients, as demonstrated by Costello et al., and may contribute to poor treatment adherence if not taken into account.^12^

Cushing syndrome is characterized by classic clinical findings involving different metabolic disorders caused by GC: central obesity, body fat redistribution, buffalo hump, moon facies, acne, striae, muscle weakness, fatigue, arterial hypertension and insulin resistance.^1^, ^7^ Its occurrence is directly associated with the duration and dosage of GC use, especially prednisone doses > 7.5 mg/day. Its management is based on the reduction of dosage and treatment duration.^7^

## Adrenal insufficiency

GCs are the final mediators of the hypothalamic-pituitary-adrenal axis. When in excess, they exert a negative feedback effect on the paraventricular nucleus of the hypothalamus and the anterior pituitary gland, causing a reduction in the secretion of corticotropin-releasing hormone and adrenocorticotropic hormone. As a result, there is atrophy of the zona reticularis and zona fasciculata of the adrenal gland, with reduced release of cortisol and androgens.^13^

The risk of adrenal suppression should be considered in patients using equivalent daily doses of prednisone ≥ 20 mg for ≥ 3 weeks, although the adrenal response to GC use may vary between patients.^7^ Corticoid therapy with nocturnal use and fractionated daily doses also seem to be risk factors for adrenal insufficiency.^7^, ^13^ Abrupt withdrawal or rapid GC tapering may cause symptoms of adrenal insufficiency (GC withdrawal syndrome): asthenia, fatigue, nausea, vomiting, diarrhea, abdominal pain, fever, arthralgia, myalgia, and weight loss. In more severe cases, hypotension, lethargy, decreased level of consciousness, seizures, hypoglycemia and coma (adrenal crisis) may occur.^7^

To prevent adrenal insufficiency, GC treatment for the patient's underlying disease should be established at the lowest effective daily dose and with the shortest possible duration. However, tapering can be a challenge due to patient's individual characteristics and mainly due to the lack of data in the literature to guide a standardized tapering strategy.^7^, ^13^ Tapering must be individualized according to clinical judgment and the patient's evolution. In cases of active disease, tapering should be slower.^7^ In patients using dexamethasone, the GC must be converted to an equivalent dose of prednisone before initiating the tapering, which can be managed with or without the aid of morning serum cortisol measurement. Prete recommends that, in patients using GCs with doses > 40 mg/day, rapid tapering can be performed with a weekly reduction of 5‒10 mg of the daily dose, until reaching the dose of 20 mg/day; with doses between 20 and 40 mg/day, the weekly reduction should be 5 mg of the daily dose until reaching the dose of 20 mg/day; when the dose is between 10 and 20 mg/day, tapering should be slower, with a recommended weekly reduction of 1‒2.5 mg of the daily dose until reaching the dose of 10 mg/day; with doses between 5 and 10 mg/day, a weekly reduction of 1 mg in the daily dose is recommended, until reaching a dose of 5 mg/day; upon reaching this daily dose, discontinuation should only be carried out after adrenal function recovery is documented with a laboratory test in the morning after 24 hours without GC use.^13^ If adrenal recovery cannot be documented through laboratory testing, it is recommended that once the 5 mg/day dose is reached, the daily dose be reduced by 1 mg weekly until discontinuation.^7^ All these recommendations are summarized in Table 2.

**Table 2:** Recommendations for glucocorticoid tapering according to the daily dose of prednisone.

≥ 40 mg/day:	Weekly reduction of 5‒10 mg in the daily dose until reaching 20 mg/day.
20‒40 mg/day:	Weekly reduction of 5 mg in the daily dose until reaching 20 mg/day.
10‒20 mg/day:	Weekly reduction of 1‒2.5 mg in the daily dose until reaching 10 mg/day.
5‒10 mg /day:	Weekly reduction of 1 mg in the daily dose until reaching 5 mg/day.
≤ 5 mg/day:	Withdrawal only with adrenal function recovery documented by laboratory test in the morning, after 24 hours without the use of corticosteroids.^a^ In the absence of the test, weekly reduction of 1 mg in the daily dose until complete withdrawal.^b^
Recommendations:	• Individualize tapering according to patient's characteristics and disease.
	• Instruct the patient about adrenal insufficiency symptoms.
	• If symptoms of adrenal insufficiency occur, interrupt tapering for 2‒4 weeks. Consider support from an endocrinologist.

a Prete et al.^13^

b Caplan et al.^7^

The patient should be educated about adrenal insufficiency signs when starting the tapering. If this occurs, tapering must be stopped immediately and the GC dose can be increased or the patient can temporarily receive hydrocortisone to improve symptoms. Tapering can be resumed after 2 to 4 weeks, with the support of an endocrinologist.^7^ New modified-release GCs that mimic circadian cortisol metabolism have been tested and may represent a future alternative to prevent iatrogenic adrenal insufficiency.^13^

## Osteoporosis

GCs interfere with bone tissue health, both directly and indirectly. In excess, they inhibit osteoblastogenesis and stimulate osteoblast and osteocyte apoptosis. Additionally, they increase bone resorption by stimulating osteoclast activity. Indirect effects on bone metabolism include inhibition of the IGF1 hormone (bone formation stimulant) and calcium homeostasis imbalance, through reduced intestinal absorption and increased renal excretion of calcium, which leads to secondary hyperparathyroidism, and stimulates bone reabsorption.^14^, ^15^ Thus, the chronic use of GCs drastically reduces bone mass in trabecular bones, mainly vertebral bodies, favoring the occurrence of osteoporosis and bone fractures.^1^, ^16^ Today, it is considered the main iatrogenic cause of osteoporosis; it may occur in up to 50% of patients on chronic corticosteroid therapy, and risk of fractures may increase by up to 75% after the first three months of GC use.^3^

Both the cumulative dose and duration are associated with the risk of bone fractures. However, doses as low as 5 mg/day of prednisolone for a long term have also been associated with an increased risk of vertebral and hip fractures.^1^ Other important risk factors for GC-induced osteoporosis found in the literature are: advanced age, female gender, low Body Mass Index (BMI), low Bone Mineral Density (BMD), previous fractures, falls, smoking, alcohol abuse, family history of osteoporosis and hypovitaminosis D.^3^, ^4^

Every patient starting treatment with GCs expected to last for ≥3 months should be advised about the risk of osteoporosis and undergo clinical screening ^3^ It is recommended to investigate the previously mentioned risk factors, annually assess bone mineral density through densitometry, annually assess serum levels of 25-hydroxy-vitamin D, calcium and creatinine, and assess fracture risk.^3^, ^14^ After the age of 40, fracture risk can be assessed using the Fracture Risk Assessment Tool (FRAX), a tool available online that estimates the 10-year probability of a patient suffer a fracture based on different risk variables.^3^

As a preventive measure, it is essential that patients have an adequate daily intake of calcium and vitamin D. The American College of Rheumatology recommends an intake of 1000‒1200 mg/day of calcium and 600‒800 IU/day of vitamin D.^17^ This group recommends that the serum level of 25-hydroxy-vitamin D be maintained >20 ng/mL, although other societies recommend levels >30 ng/mL.^17^, ^18^ The patient should also be encouraged to have a balanced diet, stop smoking, reduce alcohol consumption, and engage in physical activity with weights.^17^

Oral bisphosphonates (alendronate, risedronate) are the main drugs of choice for the treatment of GC-induced osteoporosis, alongside calcium and vitamin D supplementation.^17^, ^18^, ^19^ In the case of chronic GC use, the American College of Rheumatology recommends their prophylactic use in specific populations and according to the fracture risk determined by FRAX: adults aged ≥ 40 years (women with no childbearing potential and men) with moderate to high fracture risk should use bisphosphonates; adults aged <40 years (women with no childbearing potential and men) with a previous fracture due to osteoporosis or using GC at a dose ≥7.5 mg/day of prednisone for ≥6 months and with a Z score <-3 or bone loss ≥10%/year, should also use bisphosphonates.^17^ They must be taken on an empty stomach and, if they are not tolerated due to gastrointestinal effects, they can be replaced by intravenous zoledronic acid. When indicated, bisphosphonates should be taken continuously during the GC treatment and a “drug holiday” (planned interruption in the continuous use of the medication) is not recommended.^3^ They should be avoided in women with childbearing potential, due to risk of fetal malformations, and when creatinine clearance is < 30 mL/min. Osteonecrosis of the mandible and atypical femur fracture are two rare complications of these medications, but they should always be remembered by the physician when prescribing them.^3^, ^14^

When there are contraindications to the use of bisphosphonates, other medications have been shown to be effective for the preventive treatment of osteoporosis, such as teriparatide, RANK-L inhibitors (denosumab), and calcitonin.^3^, ^14^, ^20^, ^21^ It is recommended that the prescription of these medications be carried out jointly with a professional who is specialized in the treatment of bone disorders.^3^

The chronic use of GCs is also considered to be one of the main causes of non-traumatic osteonecrosis. It occurs mainly in the distal femur and proximal tibia.^3^ The mechanism for its occurrence is not fully understood, but it seems to be due to structural bone collapse secondary to osteocyte apoptosis, vascular thrombosis, fat embolism and stress fractures. It usually manifests with joint pain and its main risk factor is prolonged use of GCs at high cumulative doses. Therefore, the patient should always be asked about this symptom. Such complications can occur in up to 40% of these patients and treatment is essentially surgical, associated with the use of bisphosphonates, aiming to increase BMD.^1^, ^3^ Table 3 displays the main considerations for GC-induced osteoporosis.

**Table 3:** Main risk factors, prophylaxis and monitoring for glucocorticoid-induced osteoporosis.

Main risk factors:	Dose/duration of treatment, advanced age, female gender, low BMI, low BMD, previous fractures, falls, smoking, alcohol abuse, family history of osteoporosis and hypovitaminosis D.
Prophylactic measures:	• Healthy diet, physical activity with weights, reduce alcohol consumption.
	• Daily intake of calcium (1,000‒1,200 mg/day) and vitamin D (600‒800 IU/day) for all patients.
	• Associate bisphosphonates in specific cases.^a^
	• Assess fracture risk (FRAX).
Monitoring:	• Assess serum 25-OH-vitamin D, creatinine and calcium levels annually.
	• Annual bone densitometry.
	• Always inquire about joint pain.
	• Consider support from an osteoporosis specialist.

^a^ Indication of prophylactic use of bisphosphonates:

• Adults ≥ 40 years (men and women with no childbearing potential): moderate to high fracture risk.

• Adults < 40 years old (men and women with no childbearing potential): previous fracture due to osteoporosis; on glucocorticoids at a dose ≥ 7.5 mg/day of prednisone for ≥ 6 months and with a Z score < -3 or bone loss ≥ 10%/year.

BMI: Body Mass Index; BMD: Bone Mineral Density; FRAX: Fracture Risk Assessment Tool.

## Cardiovascular changes

GCs may increase the risk of cardiovascular disease due to different pathophysiological processes. These drugs have prothrombotic properties and increase the circulation of free lipids (hyperlipidemia), favoring ischemic events.^5^, ^22^, ^23^ They also cause changes in vascular tone due to an imbalance between vasoconstrictor and vasodilator substances, and increase water and sodium retention by activating mineralocorticoid receptors. Therefore, they lead to arterial hypertension through different mechanisms.^1^, ^24^ GCs can cause changes in cardiomyocytes with cardiac remodeling through activation of the renin-angiotensin-aldosterone pathway. In the long term, they induce asymmetrical thickening of the left ventricular wall and the diagnosis of heart failure.^25^

In the literature, the main risk factors for cardiovascular disease associated with the use of GCs are advanced age, male gender, obesity, pre-existing hypertension/DM/dyslipidemia, and active inflammatory disease.^2^, ^4^

The risk of hypertension may be two-fold higher in patients using GCs, regardless of treatment duration. Its incidence can reach 37% in patients over 65 years of age using high doses of GC for more than three months. A large retrospective study with a cohort of patients with rheumatoid arthritis identified a 17% increase in the risk of hypertension after starting a dose ≥ 7.5 mg/day of prednisolone.^26^ Literature data are not clear about the best drug for treatment of GC-induced hypertension. The use of medications that act on vascular resistance, such as thiazide diuretics, is recommended.^22^, ^24^

The risk of coronary disease, ischemic heart disease, heart failure, and sudden death have been associated with a two-to-four-fold increase in individuals receiving prednisolone doses ≥ 7.5 mg/day.^1^ These risks also seem to be associated with iatrogenic Cushing syndrome.^22^ Arrhythmias have been detected shortly after pulse therapy with GCs in patients with pemphigus vulgaris, which demonstrates the great variability of cardiovascular alterations attributed to these drugs.^27^

All patients using GCs should be properly educated about increased cardiovascular risk and encouraged to adopt healthy lifestyle habits. Hypertension and hyperlipidemia should be managed according to the main guidelines. There is no consensus regarding the monitoring of the lipid panel in patients undergoing chronic corticosteroid therapy. Caplan et al. recommend a biannual assessment.^22^

## Dermatological changes

The skin is also an important organ affected by chronic GC use. Skin thinning occurs due to inhibition of keratinocyte proliferation and production of collagen and hyaluronic acid by dermal fibroblasts. Lacerations, wide purple striae, telangiectasias and ecchymosis/hematomas are expected complications. Dermatoporosis is a failure in the skin barrier associated with atrophy and fragility, also compromising wound healing. Its incidence reaches 5% in patients who use GCs for more than one year, even at low doses (< 5 mg/day of prednisone). Under high doses, acne, hirsutism, and hair loss may also be observed.^1^ Therefore, it is believed that management should be directly influenced by the dose and duration of corticosteroid therapy.

This review found no studies or guidelines to guide the prevention of these complications. Since these are adverse effects directly related to dose and duration, treatment with GCs should be as short as possible and at the lowest effective dose. In the authors' clinical routine, dermatoporosis management is based on basic orientation regarding skin barrier protection, and acne/acneiform eruptions, when present, are treated according to specific guidelines.

## Muscle changes

In skeletal muscle, GCs are responsible for decreasing insulin action due to increased molecular resistance to the hormone. They also interfere with protein synthesis and stimulate muscle catabolism, which induces a state of tissue atrophy.^1^, ^5^ GCs are the leading cause of medication-induced myopathy which is characterized by painless muscle weakness, followed by atrophy, initially in the proximal musculature of the lower limbs.^1^, ^22^

The risk of GC-induced myopathy increases with treatment dose and duration, although these variables are not well studied in the literature.^22^ Fluorinated GCs (dexamethasone, betamethasone) pose a higher risk for this complication and therefore should be avoided or replaced.^1^, ^22^

Electroneuromyography findings are nonspecific and creatinine-kinase levels are normal. For clinical management, patients undergoing chronic use of GCs should always be asked about muscle weakness and, if possible, proceed to tapering.^1^, ^22^ In addition to electroneuromyography, different tests have been studied to investigate GC-induced myopathy. However, there are no specific guidelines for their use and, therefore, patients should be investigated by a multidisciplinary team.^28^ Muscle strength recovery usually occurs three to four weeks after GC discontinuation.^1^ Physiotherapy is also recommended.^22^

## Changes in the gastrointestinal tract

The use of GCs is considered a risk factor for gastrointestinal adverse events, such as gastritis, peptic ulcer, and gastrointestinal bleeding, although there are conflicting data in the literature establishing their risk when used as monotherapy.^1^, ^7^ However, when associated with non-steroidal anti-inflammatory drugs (NSAIDs), the risk of gastrointestinal ulcers and bleeding increases considerably.^7^, ^29^ It has been postulated that GCs may be associated with an increased risk for pancreatitis, but data from the literature are inconclusive.^7^

Prophylaxis for gastrointestinal complications is recommended for all patients on combined GC therapy with NSAIDs, and proton-pump inhibitors are preferred as the first choice.^7^, ^30^ When on GC monotherapy, prophylaxis should be used if the patient has risk factors (previous peptic ulcer, smoking, alcoholism, age >65 years, use of bisphosphonates). Patients should always be educated about symptoms for clinical suspicion of an adverse event and must always be referred to a gastroenterologist if they manifest signs of gastrointestinal bleeding.^7^

## Ophthalmological changes

Chronic use of GC is known to be associated with the occurrence of important ophthalmological changes. Glaucoma and cataracts are the most frequent ones.^1^ There are also reports that GC causes central serous chorioretinopathy, ptosis, mydriasis, and exophthalmos.^1^, ^22^, ^31^

The risk of ocular hypertension and glaucoma is considerably higher in patients chronically using topical, intraocular, or periocular GCs when compared to systemic GCs.^32^ However, patients on systemic use of GCs should receive adequate attention, as the risk of increased ocular pressure is estimated at 18%‒36% in this population. It may occur asymptomatically in a matter of months to years and, if not treated, can cause vision loss due to irreversible damage to the optic nerve.^1^, ^22^, ^32^ A genetic predisposition for the development of glaucoma is assumed.^1^

Cataract, usually posterior subcapsular, is associated with chronic use of systemic GCs, and its incidence can reach 58%.^22^, ^31^ There is no well-established correlation between cataract risk and dose/duration of GC treatment, but there seems to be an increased susceptibility for some individuals.^31^

Before initiating corticosteroid therapy, the patient should be asked about personal and family history of glaucoma and cataracts. An initial ophthalmologic evaluation is also recommended. It is important to reinforce the need for DM screening and treatment in order to minimize ophthalmological complications from GC use.^22^

## Neuropsychiatric disorders

It is well-recognized that GC can cause important neurological and psychiatric disorders. Mood swings, depression, euphoria, emotional lability, mania/hypomania, akathisia, attention deficit, confusion, psychosis and panic disorder are possible complications associated with GC use and are most frequent in the first three months of treatment.^1^, ^22^ These complications seem to be associated with moderate to high daily doses, prolonged treatment, and pre-existing psychiatric conditions.^22^ A large Danish population study demonstrated a possible correlation between GC use and the diagnosis of early-onset schizophrenia in the young population.^33^ The hippocampus is a brain area rich in steroid receptors and, therefore, may be vulnerable to the action of chronic corticosteroid therapy. Insomnia, memory deficit, and cognitive impairment are possible adverse events and should not be ignored by the clinician.^1^

GC-induced psychosis is a more serious complication manifested as psychosis, dementia, delirium, and suicidal ideation. It can have an early onset, days to weeks after starting GC therapy, is associated with high dosage, and can also occur during tapering.^1^, ^22^ It has been reported that women are more likely to develop depression, while men are more likely to develop mania.^22^

All patients, especially younger ones, should be asked about their neuropsychiatric history before starting treatment with GCs. It is also important to inquire about self-harming behavior or suicidal ideation.^22^ In over 90% of cases, symptoms resolve six weeks after discontinuing GC therapy.^1^ Patients should always be referred to a specialist if major adverse effects occur. Referral should also be recommended for patients with previous neuropsychiatric conditions who will undergo chronic corticosteroid therapy.^22^

## Infections and vaccines

One of the main effects of GCs is immunosuppression, which is widely used to control systemic autoinflammatory diseases. However, this effect poses a risk of vulnerability to every patient, as a state of systemic immunosuppression favors the emergence and reactivation of several infections.^1^, ^34^ GCs can interfere with virtually all cells of the immune system. They antagonize macrophages and suppress the production of the main pro-inflammatory cytokines; they suppress endothelial adhesion of neutrophils; inhibit the activation of T-cell subpopulations, causing significant lymphopenia; and also interfere with dendritic cell maturation and activation.^34^ As a consequence, GCs also reduce patients' vaccine response and make them prone to infections secondary to vaccination with live agents.^35^ Fardet et al. conducted a large population-based cohort study that showed a two-to-four-fold increased risk of bacterial, viral, and fungal infections when using GCs. This risk increased with age and was higher in patients with DM, low serum albumin levels, and those using high doses of GCs.^36^ There are reports that also associate GCs with opportunistic eye infections, such as herpetic keratitis and cytomegalovirus retinitis.^1^

In this review, the main infectious diseases reported in the literature, emphasizing the need for their screening and prophylaxis, were: pneumocystis pneumonia (PCP), tuberculosis (TB), HIV/AIDS, hepatitis B and C, strongyloidiasis and herpes zoster. The considerations related to preventive measures for these infections were didactically summarized in Table 4.

**Table 4:** Recommendations for infections screening, prophylaxis and vaccination in chronic systemic corticosteroid therapy.

Pre-treatment screening:	• Serological tests: Anti-HIV, HBsAg, Anti-HBc, Anti-HCV.
	• Consider TB test (Mantoux or IGRA test) if population at risk.^a^
Prophylactic measures:	• PCP: consider prophylaxis if there are other risk factors (hematological malignancy, interstitial lung disease, use of another immunosuppressive drug). Drug options: trimethoprim-sulfamethoxazole 800/160 mg/day (every day or 3 times/week) or dapsone 100 mg/day or atovaquone (1500 mg/day).
	• Strongyloidiasis: consider prophylaxis if the patient is from an endemic area. Drug options: ivermectin 200 mcg/kg/day for 2 days or albendazole 400 mg/day for 3 days.
Vaccination:	• Confirm that the patient is up to date with the following vaccines: *H. influenza* B, hepatitis A and B, influenza, HPV, *N. meningitides*, measles, mumps, rubella, tetanus and *S. pneumoniae*. If not, vaccinate 2‒4 weeks before treatment.
	• Varicella zoster: vaccinate patients >50 years, 2‒4 weeks before treatment.
	• Vaccinate against influenza annually.
	• Avoid vaccines with live agents.

^a^ Population at risk for TB: patients with previous intimate contact with individuals with TB, prolonged exposure in prisons or health facilities, substance abusers and inhabitants of endemic areas.

TB: Tuberculosis; IGRA: Interferon Gamma Release Assay; PCP: Pneumocystosis Pneumonia; HPV: human papillomavirus.

## PCP - Pneumocystosis Pneumonia

It is an opportunistic infection most commonly diagnosed in patients with HIV/AIDS. Although it may occur in immunosuppressed patients due to other causes, there is no data in the literature to guide screening or prophylaxis in individuals receiving high doses of GCs. In patients on an equivalent dose of prednisone ≥20 mg/day for ≥four weeks, PCP prophylaxis is recommended if there is another associated risk factor (hematological malignancy, interstitial lung disease, use of another immunosuppressive drug). For prophylaxis, there is a predilection for the combination sulfamethoxazole-trimethoprim at a daily dose of 800/160 mg (double-strength), every day or three times a week. As an alternative to trimethoprim-sulfamethoxazole, dapsone (100 mg/day) or atovaquone (1,500 mg/day) can also be used.^34^, ^35^

### Tuberculosis

The chronic use of GCs may favor the reactivation of tuberculosis in previously exposed patients. The dose and duration related to the risk of this disease are still unclear, but this correlation has already been made with prednisone doses ≥15 mg/day for more than one month.^34^, ^35^ There is also no specific recommendation in the literature for TB screening when starting GC treatment.^34^ In general, TB screening using tuberculin tests is recommended in populations considered at risk, so they can receive adequate treatment and don't reactivate the bacillus during GC treatment. The following are considered at-risk populations: patients with previous intimate contact with individuals with TB, prolonged exposure in prisons or health units, substance abusers and inhabitants of endemic areas. However, some authors recommend always performing the screening before starting corticosteroid therapy.^35^ The tuberculin skin test (Mantoux reaction) in immunosuppressed patients should be considered positive when ≥ 5 mm. However, the test may be false negative when using GC doses ≥15 mg/day for ≥two to four weeks. Another screening alternative is the use of the Interferon Gamma Release Assay (IGRA), which seems to suffer less interference from GC use.^34^ Every patient with a positive screening should be investigated with a chest X-ray and sputum analysis to confirm whether the infection is active or latent, and should also be referred to an infectious disease specialist.^35^ In the authors clinical practice, they do not perform TB screening in all patients before corticosteroid therapy. Therefore, clinical judgment is essential to determine screening, especially in at-risk populations.

### HIV/AIDS

All patients undergoing chronic treatment with GC must be tested for HIV. If undiagnosed or untreated, the use of GCs greatly increases the risk of infections in these patients.^35^

### Hepatitis B and C

Due to the risk of increased viremia, screening for hepatitis B (HBsAg, anti-HBs and anti-HBc) and hepatitis C (anti-HCV) is recommended in every patient who will use GCs for a long period, particularly with prednisone doses ≥ 20 mg/day for more than four weeks.^35^

### Strongyloidiasis

This is a chronic parasitic disease caused by *Strongyloides stercoralis*, which is usually endemic in tropical and subtropical countries. This parasite is capable of causing self-infection and, therefore, can remain in the host indefinitely.^34^, ^35^ In immunosuppressed individuals, it can cause a severe condition called hyperinfection, whose mortality ranges from 15% to 87%. It manifests with a variety of signs/symptoms: abdominal pain, nausea, vomiting, diarrhea, intestinal obstruction, gastrointestinal bleeding, coughing, hemoptysis, edema, ascites, and Gram-negative bacteremia. When disseminated, it can even cause dermatological manifestations (thumbprint sign).^35^, ^37^ Clinical suspicion can be overlooked due to the heterogeneity of symptoms, and stool analyses can often come back negative. Anemia, hypereosinophilia and hypoalbuminemia are suggestive laboratory findings. Among the therapeutic options, ivermectin is the anthelmintic of choice.^37^ In endemic areas, many clinicians recommend empiric treatment with ivermectin before starting chronic GC therapy.^35^ In the authors clinical practice, located in an endemic area, prophylaxis is carried out with ivermectin 200 mcg/kg/day for two consecutive days, or albendazole 400 mg/day for three consecutive days, always before starting immunosuppressive doses of GCs. This measure is repeated every six months.

### Herpes zoster

Vaccination for varicella-zoster is indicated for the entire population over 60 years of age, regardless of previous occurrence of herpes zoster and chronic corticosteroid therapy. In patients who are immunosuppressed by GCs, the varicella-zoster virus may reactivate more frequently and the infection may be more severe or widespread, requiring hospitalization.^35^ Therefore, it is strongly recommended that these patients receive the vaccine two to four weeks before starting GC treatment. Some authors recommend that patients over 50 years old should receive the vaccine if undergoing immunosuppressive treatment.^34^, ^35^

### Vaccination

Caplan et al. summarize the main recommendations for vaccinating individuals using GCs, based on the guidelines of large expert societies. Patients who will receive doses of prednisone ≥ 20 mg/day for more than two weeks should have their vaccination history evaluated to ensure that they are up to date with the following vaccines: *Haemophilus influenza* B, hepatitis A and B, influenza, human papillomavirus (HPV), *Neisseria meningitidis*, measles, mumps, rubella, tetanus and *Streptococcus pneumoniae*. A minimum of two to four weeks should be allowed before initiating GCs after vaccination with live agents. When vaccinated with inactive agents, this period is not indicated. However, the risk of suboptimal vaccine response is still considerable if the individual starts treatment with moderate to high doses of GCs. The influenza vaccine should be administered annually to all patients on chronic corticosteroid therapy.^35^

### Other important considerations

It has already been shown that GCs compromise the natural response to physiological stress after a surgical procedure. Moreover, they interfere with healing and increase susceptibility to infections. Chouairi et al. performed an observational study that analyzed post-surgical adverse effects in more than 180,000 GC users. In this study, it was concluded that patients using GCs who undergo a surgical procedure are at greater risk of surgical wound dehiscence, surgical site infection, and need for reintervention. The authors reinforce that there is no consensus regarding the dose or duration of treatment that interferes in the postoperative period, nor the time required after suspension of GCs use to prevent such complications. Hence, the surgeon should always be cautious when prescribing an elective procedure for this particular patient.^23^

GCs have important anti-inflammatory properties, often useful in the treatment of cancer patients. However, it is questioned whether their immunosuppressive action would not be a risk factor for the emergence of neoplasms. Cairat et al. conducted a large cohort study of postmenopausal women who were on chronic GC use to assess the impact on breast cancer. It was observed that GC use decreased the risk for stage 1/2 and invasive cancers that were estrogen-receptor positive. However, it proved to be a risk factor for *in situ* and stage 3/4 tumors. It is possible to infer, therefore, that in the context of cancer, molecular subtypes and clinical staging may present different correlations with chronic GC use.^38^

## Conclusion

The use of GCs was, in fact, one of the greatest milestones in medicine, enabling the treatment of numerous diseases and ensuring increased patient survival and quality of life. To date, their use is recommended in different guidelines for different conditions, such as autoimmune diseases, infections, and neoplasms. However, since the discovery of their therapeutic benefits, their adverse effects have also been well-known and studied, especially on chronic use. In Brazil, access to other immunosuppressive or immunobiological drugs that spare GC use is limited. This constitutes an important public health problem, also present in other countries, and can compromise the choice of the ideal treatment for a patient, placing the physician in a situation where GCs are the only therapeutic alternative.

It is mandatory that every GC prescriber be aware of the adverse effects so that, at the very least, they can identify them as early as possible. Their management often demands a multidisciplinary approach and, therefore, the dermatologist must know how to refer their patients to the most appropriate specialist whenever possible. Besides, it is also important to educate the patient and adopt the best prophylactic measures for each GC-related complication. This narrative review sought to bring together the main adverse effects of chronic systemic corticosteroid therapy, their main risk factors and measures for screening, prophylaxis and clinical monitoring. Therefore, the authors believe this review can be used as a quick learning tool and reference for dermatologists, and expect it also prompts the development of other studies and guidelines focused on the chronic use of GCs.

## Financial support

None declared.

## Authors' contributions

Lucas Campos Prudente Tavares: Design and planning of the study; drafting and editing of the manuscript; collection, analysis and interpretation of data.

Lívia de Vasconcelos Nasser Caetano: Design and planning of the study; drafting and editing of the manuscript; collection, analysis and interpretation of data; critical review of the manuscript; approval of the final version of the manuscript.

Mayra Ianhez: Critical review of the manuscript; approval of the final version of the manuscript.

## Conflicts of interest

None declared.
